# Does Impairment of Adult Neurogenesis Contribute to Pathophysiology of Alzheimer's Disease? A Still Open Question

**DOI:** 10.3389/fnmol.2020.578211

**Published:** 2021-01-22

**Authors:** Domenica Donatella Li Puma, Roberto Piacentini, Claudio Grassi

**Affiliations:** ^1^Department of Neuroscience, Università Cattolica del Sacro Cuore, Rome, Italy; ^2^Fondazione Policlinico Universitario A. Gemelli IRCCS, Rome, Italy

**Keywords:** neural stem cells, adult neurogenesis, amyloid-beta protein, tau, Alzheimer's disease, herpes simplex virus type 1

## Abstract

Adult hippocampal neurogenesis is a physiological mechanism contributing to hippocampal memory formation. Several studies associated altered hippocampal neurogenesis with aging and Alzheimer's disease (AD). However, whether amyloid-β protein (Aβ)/tau accumulation impairs adult hippocampal neurogenesis and, consequently, the hippocampal circuitry, involved in memory formation, or altered neurogenesis is an epiphenomenon of AD neuropathology contributing negligibly to the AD phenotype, is, especially in humans, still debated. The detrimental effects of Aβ/tau on synaptic function and neuronal viability have been clearly addressed both in *in vitro* and *in vivo* experimental models. Until some years ago, studies carried out on *in vitro* models investigating the action of Aβ/tau on proliferation and differentiation of hippocampal neural stem cells led to contrasting results, mainly due to discrepancies arising from different experimental conditions (e.g., different cellular/animal models, different Aβ and/or tau isoforms, concentrations, and/or aggregation profiles). To date, studies investigating *in situ* adult hippocampal neurogenesis indicate severe impairment in most of transgenic AD mice; this impairment precedes by several months cognitive dysfunction. Using experimental tools, which only became available in the last few years, research in humans indicated that hippocampal neurogenesis is altered in cognitive declined individuals affected by either mild cognitive impairment or AD as well as in normal cognitive elderly with a significant inverse relationship between the number of newly formed neurons and cognitive impairment. However, despite that such information is available, the question whether impaired neurogenesis contributes to AD pathogenesis or is a mere consequence of Aβ/pTau accumulation is not definitively answered. Herein, we attempted to shed light on this complex and very intriguing topic by reviewing relevant literature on impairment of adult neurogenesis in mouse models of AD and in AD patients analyzing the temporal relationship between the occurrence of altered neurogenesis and the appearance of AD hallmarks and cognitive dysfunctions.

## Hippocampal Neurogenesis and Memory

The hippocampus is recognized as a brain area primarily involved in memory formation, e.g., pattern separation, emotional memory, and cognitive flexibility (Lazarov and Hollands, 2016; Anacker and Hen, 2017; Hainmueller and Bartos, 2020). The hippocampal circuitry consists of a unidirectional, trisynaptic excitatory pathway in which the dentate gyrus (DG) of the hippocampus receives inputs from the entorhinal cortex (EC), which are then delivered to the CA3 area of the hippocampus and from there to CA1. In turn, CA1 projects to the subiculum and hippocampal outputs are sent back to the deep layers of EC. The classical experimental paradigm used to investigate hippocampal plasticity, underlying memory formation, is the long-term potentiation (LTP) at the CA3-CA1 synapse not involving the DG (Malenka and Nicoll, 1999), and the EC-DG-CA3-CA1 axis could be strongly affected if this pathway is corrupted at the DG. In fact, DG supports various mnemonic functions including contextual discrimination, pattern separation, novelty detection, and integration of individual episodes into a framework of experiences (Hainmueller and Bartos, 2020). Interestingly, the subgranular zone (SGZ) of the DG in the hippocampus is known to be one of the two neurogenic niches in the adult brain, the other being the subventricular zone of the lateral ventricles (Gage, 2019). Adult neurogenesis occurring at the SGZ allows integration of newly formed neurons in the DG circuits (Kitabatake et al., 2007), thus providing this brain area with marked plasticity. As such, adult hippocampal neurogenesis at the DG has been proposed to strongly participate in formation of hippocampal-dependent memory (Gonçalves et al., 2016; Toda et al., 2019; Hainmueller and Bartos, 2020).

Additionally, the hippocampus is one of the brain areas primarily affected by aging and Alzheimer's disease (AD) (Selkoe, 2011). Alzheimer's disease is the most common cause of dementia in the elderly and is characterized by memory loss and cognitive dysfunction. The majority of AD cases are sporadic, and the remaining cases are associated with genetic factors [i.e., familial AD (FAD)]. Mutations in genes, encoding either the amyloid precursor protein (APP) or enzymes catalyzing its proteolytic cleavage (presenilin 1 and 2—PSEN1 and 2—subunits of γ-secretase responsible for amyloid-β peptide—Aβ–generation), along with microtubule-associated protein tau (MAPT) encoding the tau protein, are responsible for FAD. Nowadays, it is widely recognized that memory failure in AD is due to synaptic alterations caused by intra- and extracellular accumulation of Aβ and hyperphosphorylated tau oligomers (Crews et al., 2010; Puzzo et al., 2017). Experimental evidence also suggests that early alterations of DG neurogenesis may concur to the pathogenesis of this neurological disorder (Mu and Gage, 2011; Unger et al., 2016).

## AD and Neurogenesis: *in vitro* Studies

In the last 15 years, many studies investigated hippocampal neurogenesis [i.e., proliferation and neuronal differentiation of hippocampal neural stem cells (NSCs)] in *in vitro* and *in vivo* experimental AD models.

*In vitro* experimental paradigms usually consisted of NSC incubation with Aβ. However, researches carried out in various laboratories led to contrasting results about the effects of proliferation/differentiation of hippocampal NSCs, mainly because of different (i) Aβ preparations (monomeric, oligomeric, or fibrillar); (ii) peptide lengths (40 vs. 42 amino acids); and (iii) working concentrations, which are often irrelevant from a physiological point of view. For example, in 2004 López-Toledano and Shelanski reported that *in vitro* treatment of hippocampal NSCs with micromolar concentrations of Aβ42 oligomers dose-dependently increased their neuronal differentiation (López-Toledano and Shelanski, 2004). Conversely, more recent results obtained either with lower Aβ concentrations or in cultured NSCs isolated from mouse models harboring the most common genetic alterations observed in FAD indicated impaired proliferation and reduced neuronal differentiation of hippocampus-residing NSCs. In particular, Lee et al. (2013) found that exposure of human NSCs to Aβ-containing conditioned medium from SK-N-MC cells expressing APP Swedish mutation reduced NSC proliferation, impaired neurogenesis, and promoted gliogenesis via glycogen synthase kinase 3β (GSK-3β) activation. Moreover, the exposure of human neural stem cell line hNS1 to nanomolar concentrations of Aβ42 significantly promoted cell proliferation and glial cell specification by increasing the pool of proliferating glial precursors without affecting neuronal differentiation (Bernabeu-Zornoza et al., 2019). A recent study from our lab demonstrated that treatment of cultured murine hippocampal NSCs with Aβ42 oligomers (200 nM)—able to cross plasma membrane, to accumulate intracellularly, and to induce GSK-3 activation (Ripoli et al., 2013, 2014, Scala et al., 2015)—negatively affected their proliferation and neuronal differentiation (Li Puma et al., 2019). Similar results were obtained in NSCs infected with HSV-1, which triggered APP phosphorylation and cleavage with consequent accumulation of several APP fragments including Aβ in several cell types (De Chiara et al., 2010; Piacentini et al., 2011, 2015). The HSV-1-induced hyperproduction of Aβ was correlated with the antimicrobic activity of Aβ42 and interpreted as a defensive response of the infected cell (Soscia et al., 2010; Kumar et al., 2016). Strategies aimed at limiting the production and accumulation of Aβ inside cells (as the use of a γ-secretase inhibitor or the 4G8 antibody raised against Aβ oligomers able to be intracellularly uploaded; Tampellini et al., 2007) counteracted the effects of Aβ42 on *in vitro* neurogenesis (Li Puma et al., 2019). Altered proliferation and neuronal differentiation were also observed in NSCs isolated from 3 × Tg-AD mouse embryos (Leone et al., 2019). These cells exhibited high levels of Aβ oligomers compared with NSCs isolated from wild-type (WT) mouse. Emerging evidence also suggests that downregulated expression of the nucleoporin Nup153 negatively affects the neurogenic niche of 3 × Tg AD mice. Accordingly, restoration of Nup153 levels in hippocampal 3 × Tg-AD NSCs promoted their proliferation, migration, and neuronal maturation (Leone et al., 2019).

## Hippocampal Neurogenesis in FAD Mouse Models

More robust results have been obtained from studies performed in *in vivo* FAD mouse models often exhibiting impaired neurogenesis correlated with accumulation of molecular AD hallmarks (e.g., Taniuchi et al., 2007; Hollands et al., 2016, 2017; Baglietto-Vargas et al., 2017). However, depending on the FAD model used, this impairment may rely on reduced neurogenesis (lower NSC proliferation, decreased neuronal differentiation, and/or reduced survival of newly formed neurons) or increased gliogenesis (normal of even increased NSC proliferation, followed by differentiation toward the glial rather than neuronal phenotype). Nevertheless, despite the consensus about altered neurogenesis in these mouse models, a clear understanding of whether and how much this impairment contributes to memory/cognitive dysfunction in AD is still missing.

Various FAD mouse models have been developed resembling peculiar features of the disease, which are based on one or more gene-coding mutations in proteins critically involved in AD (Unger et al., 2016). The most applied models use *Tg2576 mic*e (Hsiao, 1998), which overexpress APP harboring the Swedish double mutation—KM670/671NL; *PDAPP mice* (Games et al., 1995), which overexpress APP harboring the Indiana (V717F) mutation; and *J20 mice* (Mucke et al., 2000), which overexpress APP harboring both Swedish and Indiana mutations. Other models associate APP mutations to other mutations accounting for PSEN1 and 2 genes encoding for presenilin 1 and 2, as for example the double transgenic “*APP Swedish PS1*Δ*E9*” (APPswe/PS1ΔE9) mouse model (Jankowsky et al., 2001) or *APP/PS1 mice* harboring APP Swedish and London (V717I) along with the PS1 M146L mutation (Baglietto-Vargas et al., 2017). Finally, the 5 × FAD mouse model, which is a more complex model harboring all five AD-linked mutations accounting for Aβ formation, has also been developed (Oakley et al., 2006). All these models do not consider familial mutations involving the tau protein, which is the other key protein in AD. In this regard, a mouse model representative of the full AD pathology has been developed, associating mutation in the MAPT gene, encoding for tau protein, with those of the other key proteins in AD (APP and PS1). This 3 × Tg-AD model contains three mutations associated with FAD: APP Swedish, PSEN1 M146V, and MAPT P301L (Oddo et al., 2003).

What about neurogenesis in these FAD mouse models? Demars et al. (2010) reported a drastic reduction of NSC proliferation [identified through 5′-bromo-deoxyuridine (BrdU) incorporation] in the SGZ of the hippocampus of APPswe/PS1ΔE9 mice, at 2 months of age, with respect to the age-matched WT animals. These alterations, taking place before the formation of amyloid deposits, were followed by a significant reduction in the number of cells acquiring a neuronal (doublecortin^+^ -DCX) phenotype (i.e., BrdU^+^/DCX^+^ cells) with respect to age-matched WT mice. In agreement with Demars's results, in the same experimental model Unger et al. (2016) found a reduced number of BrdU-positive cells evaluated at 3 months of age, 30 days after BrdU injection, but an increased numbers of PCNA^+^ cells. PCNA is a protein expressed by proliferating cells in the late G1 and S phases of mitosis, and this difference, observed with these two methods of analysis, may suggest alteration in the cell cycle. However, most of these new cells (positive for either BrdU or PCNA) did not survive during maturation resulting in a reduced number of BrdU^+^/DCX^+^ and PCNA^+^/DCX^+^ cells, thus indicating impaired adult hippocampal neurogenesis. Similar findings were also obtained in Tg2576 mice, which showed increased NSC proliferation in the SGZ of the hippocampus DG but reduced integration of newly formed neurons in the DG at an age at which these mice exhibited neither amyloid extracellular deposits nor major cognitive impairment (Unger et al., 2016). Unger's data in Tg2576 mice were in agreement with those obtained by Krezymon et al. (2013) in the same mouse model. In APP/PS1 mice, Baglietto-Vargas et al. found a reduced number of SGZ precursor cells along with reduced numbers of BrdU^+^/DCX^+^ cells at 4–6 months, i.e., slightly before the onset of cognitive dysfunction (Baglietto-Vargas et al., 2017). Also, 5 × FAD mice exhibited an early impairment of neurogenesis with significantly reduced DCX^+^ cells in the DG starting from 2 months of age (Moon et al., 2014). In 2008, Rodrìguez et al. demonstrated impaired neurogenesis in terms of NSC proliferation and neuronal differentiation/integration of the DG even in 3 × Tg-AD mice. In this mouse, a significant reduction of neurogenesis was evident in females at 4 months of age with respect to age-matched controls, while male mice exhibited these alterations later. Findings about early alteration of hippocampal neurogenesis in 3 × Tg-AD mice were also confirmed by Hamilton et al. (2010). Interestingly, Zheng et al. (2017) demonstrated that intrahippocampal injection of Aβ42 in ovariectomized mice inhibited neurogenesis, which were recovered by 17β-estradiol (E2) treatment; this finding further supported the impact of estrogens in regulating neurogenesis and their potential role in AD pathogenesis. Of note, several conflicting results have been reported on the J20 mouse model, which was found to exhibit either increased neurogenesis with increasing age (López-Toledano and Shelanski, 2007) or impaired neurogenesis independently on Aβ (Pan et al., 2016) and additionally on the APP/PS1 mice, which exhibited increased neurogenesis at later age (Yu et al., 2009), thus indicating that effects on neurogenesis may also depend on a combination of mutations.

A detailed description of how neurogenesis is altered in different AD mouse models was reviewed by Chuang (2010) and Wirths (2017). In Table 1, we summarized how neurogenesis is altered in various AD models, highlighting the age at which neurogenesis was impaired and the age at which cognitive dysfunction started.

**Table 1:** Alteration of adult hippocampal neurogenesis in FAD and sporadic AD mouse models.

**Mouse model**	**Alteration in hippocampal neurogenesis**	**Age at impaired neurogenesis**	**Age at the occurrence of cognitive decline**	**Reference(s)**
APP Swe PS1 ΔE9 (FAD)	Reduced proliferation of NSCs (BrdU^+^). Reduced survival of newly generated cells. Reduced neuronal commitment (DCX^+^)	2 months	8 months	Demars et al., 2010
	Increased number of PCNA^+^ and PCNA^+^/DCX^+^ NSCs. Reduced number of BrdU^+^ NSCs 30 days after BrdU injection. Reduced number of newly generated cells neurons (BrdU^+^/NeuN^+^)	3 months		Unger et al., 2016
Tg2576 (FAD)	Increased proliferation of NSCs (PCNA^+^) and of newly generated neuroblasts (PCNA^+^/DCX^+^ cells). Reduced survival of newly generated cells. Reduced number of newly generated cells neurons (BrdU^+^/NeuN^+^)	3 months	6–8 months	Unger et al., 2016
APP/PS1 (FAD)	Reduced proliferation of NSCs (BLPL^+^). Reduced number of BrdU^+^/DCX^+^ cells	4–6 months	6 months	Baglietto-Vargas et al., 2017
	Increased proliferation of NSCs (BrdU^+^); increased number of newly generated neuroblasts (BrdU^+^/DCX^+^) and mature neurons (BrdU^+^/NeuN)	9 months	9 months	Yu et al., 2009
5xFAD (FAD)	Decrease of neuroblasts (DCX^+^) in the DG	2 months	4–5 months	Moon et al., 2014
3xTg-AD (FAD)	Reduced proliferation and neuronal differentiation	3–4 months (for female mice)	6 months	Rodríguez et al., 2008
J20 (FAD)	Increased proliferation and neuronal differentiation	3 months	4 months	López-Toledano and Shelanski, 2007
HSV-1 infected (sporadic)	Reduced proliferation of NSCs (BrdU^+^) along with reduced neuronal commitment (DCX^+^)	5 months	10 months	De Chiara et al., 2019; Li Puma et al., 2019

The finding that impairment of hippocampal neurogenesis in FAD mice occurs before (i) AD hallmarks accumulation and (ii) appearance of learning and memory dysfunction suggests that the former might have a causal role in cognitive decline characterizing prodromal AD.

In support of this hypothesis, experimental evidence indicates that ablation of hippocampal neurogenesis in APPswe/PS1ΔE9 mice alters hippocampal circuitry and excitability exacerbating performance deficits with respect to age-matched non-ablated animals (Hollands et al., 2017). Similar results were also observed in 5 × FAD mice, exhibiting worsened cognitive abilities after ablation of adult hippocampal neurogenesis (Choi et al., 2018). On the contrary, stimulation of neurogenesis with drugs (P7C3), Wnt3-expressing lentivirus, or physical exercise ameliorates cognitive deficits in transgenic 5 × FAD mice and reduces amyloid burden (Choi et al., 2018). These authors suggested that neither exercise nor stimulation of adult hippocampal neurogenesis alone had beneficial effects but only the association of the two stimuli was effective in this AD mouse model. In slight contrast with this study, physical exercise was sufficient to reduce Aβ plaque burden in 3 × Tg-AD mice, to increase neurogenesis at the DG, and to improve cognitive functions (Kim et al., 2019). Moreover, the experimental paradigm of “enriched environment” was effective in ameliorating cognitive functions in APPswe/PS1ΔE9 mice along with rescue of neural progenitor cell proliferation in the hippocampus, survival and incorporation of newly born cells in preexisting hippocampal circuits, and reduction of Aβ load and tau phosphorylation in the hippocampus of this FAD model (Lazarov et al., 2005; Hu et al., 2010). In any case, all these studies highlighted a positive correlation between hippocampal neurogenesis and cognitive functions in AD experimental models, even if it is known that physical exercise does not selectively improve neurogenesis but it acts on several targets (e.g., BDNF and other factors; Saraulli et al., 2017; Liu and Nusslock, 2018), which may support cognitive functions independently on neurogenesis. Finally, Yan et al. (2014) showed that adult bone marrow-derived mesenchymal stem cell transplantation improves memory and cognitive functions of APP/PS1 mice by enhancing endogenous neurogenesis in hippocampal SGZ. Another recent study (Micci et al., 2019) demonstrated that exosomes (containing miR-322, miR-17, and miR-485 miRNAs acting at the synaptic level), released from NSCs, significantly decrease Aβ oligomer binding at synapses and protect the hippocampus from Aβ oligomer-induced impairment of LTP and memory deficits. Therefore, NSCs might significantly contribute to fight the progression of the disease, independently on the replacement of lost neurons. Conversely, a partial rescue of the impairment of adult hippocampal neurogenesis was observed following reductions of Aβ load in double transgenic APP/PS1 mice by passive Aβ immunotherapy (Biscaro et al., 2009).

Notably, all these studies demonstrated that stimuli ameliorating/increasing neurogenesis reduce the appearance of AD hallmarks; this suggests the possibility that not only Aβ/pTau affect neurogenesis, but also molecular mechanisms controlling neurogenesis influence Aβ clearance/degradation and/or tau phosphorylation.

Even if aging does not represent a pathological condition, it is a main risk factor for AD and aged people and experimental models often exhibit a decline of cognitive abilities. Dentate gyrus is one of the primary initial targets of normal aging (reviewed in Lazarov and Hollands, 2016), and hippocampal neurogenesis is negatively affected by this process, resulting in reduced NSC proliferation rates, neuroblast numbers, and immature neurons as well as differentiated granule cells in the DG (Lazarov and Hollands, 2016; Toda et al., 2019). These aging-dependent effects on neurogenesis could impact on structural and functional plasticity of the hippocampus, likely contributing to cognitive deficits in the elderly. Indeed, strategies aimed to increase adult neurogenesis in the hippocampus of aged mice [e.g., by transient overexpression of a negative regulator of dendritic spines, Kruppel-like factor 9 (McAvoy et al., 2016) or by attenuating bone morphogenetic protein signaling (Yousef et al., 2015)] improved their cognitive abilities and long-term memory (Toda et al., 2019). Notably, the above-cited FAD models also showed an age-dependent decrease in neurogenesis associated with an increase in the number of Aβ-containing neurons in the hippocampus and the presence of Aβ plaques.

## Hippocampal Neurogenesis in HSV-1-Infected Mouse Model

All the mouse models discussed above are genetically modified to develop AD hallmarks/pathology. This does not resemble what occurs in sporadic AD, in which a “normal” subject not carrying familial AD mutations starts exhibiting signs of impaired memory and learning because of undefined triggering factors. From this point of view, data obtained in a mouse model of HSV-1 infection and the recurrent reactivation that we recently set up are worth mentioning (De Chiara et al., 2019). This mouse model is reminiscent of sporadic AD phenotype. In fact, after infection and multiple cycles of virus reactivation promoting its spreading within the brain, the infected mice exhibited accumulation of amyloid-β and hyperphosphorylated tau proteins in several brain areas including the hippocampus. These molecular changes were accompanied by memory deficits that were very marked after 7 cycles of viral reactivation in mice and not found in age-matched mock-infected mice not exhibiting Aβ or pTau accumulation (De Chiara et al., 2019).

Infected mice also exhibited impaired adult hippocampal neurogenesis consisting in (i) reduced proliferation of NSCs residing in the SGZ and (ii) decreased differentiation toward the neuronal phenotype. These changes were statistically significant before the onset of memory deficits, i.e., after 2 cycles of thermal stress. Specifically, in the brain of infected mice the number of proliferating NSCs (identified through BrdU and Ki67 labeling) in the SGZ of the DG was significantly reduced with respect to mock-infected mice, and the percentage of cells acquiring glial phenotype [i.e., immunoreactive for the glial fibrillary acidic protein (GFAP)] vs. neuronal one [doublecortin (DCX)-positive] was significantly higher than in mock-infected cells (Li Puma et al., 2019). These findings suggested us that in this experimental model alteration of hippocampal neurogenesis precedes memory impairment, strongly supporting the contention that altered neurogenesis contributes to memory deficits also in sporadic AD.

## Hippocampal Neurogenesis, APP, and APP Cleavage Products

Interestingly, mice lacking amyloid precursor protein (APP KO) did not exhibit alterations in neurogenesis following HSV-1 infection and recurrent virus reactivations (Li Puma et al., 2019) despite the presence of viral particles in the hippocampus. Rather, mice lacking APP exhibited a higher number of BrdU-positive cells in the DG (Coronel et al., 2019a; Li Puma et al., 2019). These results suggest that virus *per se* does not have direct effects on neurogenesis and that APP cleavage products (e.g., Aβ) may play a major role in modulating adult hippocampal neurogenesis. As extensively reviewed in Lazarov and Demars (2012), Aβ is not the only APP product affecting neurogenesis in the hippocampus of adult mice. Other APP metabolites, derived from proteolytic processing by specific secretases, such as the secreted N-terminal-soluble fragments of APP cleaved by α- (sAPPα) or β-secretase (sAPPβ), and the APP intracellular C-terminal domain (AICD), cleaved by γ-secretase, reportedly modulate various NSC functions including proliferation, neuronal vs. glial differentiation, and death (Coronel et al., 2019b). In particular, sAPPα, obtained by the physiological non-amyloidogenic cleavage of APP, has neuroprotective and neurotrophic functions by promoting proliferation of NSCs (Demars et al., 2011, 2013). In contrast, sAPPβ derived by the Aβ-producing amyloidogenic pathway of APP has a lower efficacy than α counterpart (Demars et al., 2013). However, both sAPPα and sAPPβ were found to increase proliferation of neural precursor cells derived from the SGZ of adult rats *in vitro* and to promote glial differentiation (Baratchi et al., 2012). Finally, in human embryonic teratocarcinoma cells (NT-2/D1), often used as experimental model to investigate neural differentiation, sAPPα promotes glial fate (Kwak et al., 2006) by stimulating bone morphogenic protein signaling (Kwak et al., 2014), which is involved in aged-associated cognitive decline.

Unlike sAPPα and sAPPβ, AICD seems to negatively impact on neurogenesis. In fact, as reviewed in Coronel et al. (2019b) and Lazarov and Demars (2012), AICD strongly inhibited proliferation of NSCs acting as transcriptional regulator. Indeed, in AICD transgenic mice proliferation and survival of progenitor cells were strongly reduced while neuronal differentiation was unaffected (Ghosal et al., 2010). Moreover, NSC differentiation reportedly depended on γ-secretase activity (Gadadhar et al., 2011).

Although HSV-1 infection in cultured hippocampal NSCs determined increased APP processing with the consequent intracellular accumulation of Aβ peptides and intranuclear accumulation of AICD, we found that the 4G8 antibody (recognizing the 17–24 sequence of Aβ) was able to completely revert the effects of HSV-1 on *in vitro* neurogenesis, suggesting that the contribution of other APP fragments (e.g., AICD) to HSV-1-induced impaired neurogenesis is negligible. However, we cannot exclude, *in vivo*, that other APP metabolites generated after HSV-1-induced APP amyloidogenic cleavage may participate in the alteration of neurogenesis.

Finally, although most of the functions exerted by APP and its proteolytic fragments have been described, the molecular mechanisms and the signaling pathways involved in these effects remain mostly unknown. APP belongs to a superfamily including the homologs APLP1 and APLP2, which are expressed in APP KO mice. While mouse KO for APP/APLP1 is viable, APP/APLP2, and APLP1/APLP2 KO mice do not survive, which stresses the importance of APLP2. Interestingly, the *in vivo* silencing of APLP2 in an APP/APLP1 double knockout mouse keeps cortical progenitors much longer in their undifferentiated state, which is consistent with the view that APLP2 plays a key role in the commitment of neuronal progenitors to neuronal differentiation (Shariati et al., 2013).

## Alzheimer's Disease, Memory Loss, and Altered Neurogenesis: The Role of Tau

Although this review primarily deals with the impact of APP fragments on neurogenesis, experimental evidence suggests that tau is the real bad player in AD. Specifically, tau oligomers target neurons and astrocytes involved in tripartite synapses, which affect synaptic transmission and synaptic plasticity (Guerrero-Muñoz et al., 2015; Fá et al., 2016; Piacentini et al., 2017; Puzzo et al., 2017; Li et al., 2018). Tau has also been reported to negatively affect hippocampal neurogenesis (see Fuster-Matanzo et al., 2012). In 2010, Demars et al. demonstrated that APPswe/PS1ΔE9 mice exhibited a significant increase in tau phosphorylation in several brain areas including the hippocampus, likely contributing to the development of the AD phenotype. As discussed above, exposure of these mice to an enriched environment reduced, besides ameliorating cognitive functions and neurogenesis, also accumulation of phosphorylated tau in their hippocampus (Hu et al., 2010). In agreement with these results, more recent studies reported that human tau mice and FTDP-17 mutant tau mice exhibited a decrease in proliferation of neuronal precursors (Komuro et al., 2015; Houben et al., 2019). Pallas-Bazarra et al. (2016) demonstrated a novel role played by tau in NSC survival after stressful stimuli; they demonstrated that tau is fundamental for morphological and functional maturation of newborn granule neurons using a tau KO mouse model. Tau^−/−^ mice show impairment in the maturation of newborn granule neurons, and they are insensitive to the modulation of adult hippocampal neurogenesis exerted by external stimuli. Tau protein also facilitates DCX-positive cell migration from the SGZ to the granular layer of the hippocampus, a process that requires a dynamic microtubule network. Therefore, it is conceivable that the increased dynamics and destabilization of microtubules caused by hyperphosphorylation of tau protein may contribute to impaired hippocampal neurogenesis (Fuster-Matanzo et al., 2009, 2012). Very recent studies reported that pTau accumulation in DG interneurons impair adult hippocampal neurogenesis by suppressing GABAergic transmission (Zheng et al., 2020) and that tau KO mice exhibit increased neurogenesis in the DG at 14 months of age compared with WT mice matched for age (Criado-Marrero et al., 2020). In support of a role for tau in neurogenesis, Houben et al. (2019) demonstrated that deletion of tau in a transgenic mouse model of tauopathy (Tg30 mice harboring FTDP-17 mutant tau) rescued the alteration in hippocampal neurogenesis exhibited by these mice.

There is a strong interplay between Aβ and tau, and a common, although controversial, opinion in the field is that tau pathology would be triggered by Aβ (Bloom, 2014). For example, it was demonstrated that endogenously produced Aβ induces tau hyperphosphorylation in cell cultures (Wang et al., 2006) and recent *in vivo* PET-imaging studies suggested that Aβ is a prerequisite for tau pathology (Franzmeier et al., 2019; Pontecorvo et al., 2019). Two main protein kinases have been shown to be involved in aberrant tau phosphorylation: the cyclin-dependent kinase (Cdk5) and GSK-3β. A deregulation of these kinases, induced by extracellular amyloid loading, results in tau hyperphosphorylation (Maccioni et al., 2001). As discussed above, tau hyperphosphorylation was found in FAD mice in which genetic alterations account for APP and its cleaving enzymes (APPswe/PS1ΔE9; Demars et al., 2010), supporting the idea of a cross talk among Aβ and tau. Reduction of Aβ burden by *scyllo*-inositol in TgF344-AD rats reduced tau pathology and rescued adult hippocampal neurogenesis (Morrone et al., 2020). We cannot exclude that the effects of tau on adult neurogenesis are mediated by Aβ load, although another view has been recently proposed that Aβ and tau exert their detrimental action by acting in parallel, probably sharing common targets, rather than acting in series (Puzzo et al., 2020).

## Alzheimer's Disease, Memory Loss, and Altered Neurogenesis: Human Studies

Despite the large number of studies about the relationship among altered neurogenesis and AD, some questions still remain unanswered: is this alteration disrupting the hippocampal circuitry involved in memory formation? Does it significantly contribute to memory loss in AD? Are the results obtained in *in vitro* and *in vivo* murine models translatable to humans? Is recovery/activation of neurogenesis a useful tool to prevent the onset and/or counteract the progression of AD? Some tentative answers to these questions can be found in recent reviews from Kempermann et al. (2018) and Cosacak et al. (2020), but more in-depth investigations are absolutely needed to address these issues.

What about neurogenesis in humans and its involvement in neurodegenerative diseases? Recently, some independent studies (Mathews et al., 2017; Boldrini et al., 2018; Moreno-Jiménez et al., 2019; Tobin et al., 2019) demonstrated that (i) adult hippocampal neurogenesis also occurs in human brain and it contributes to adding new granule cells to the DG throughout the lifespan even though the efficiency of this mechanism decreases with age and (ii) in AD patients, the number and maturation of newly generated neurons progressively decline as the disease proceeds. In particular, Tobin et al. found a significant inverse relationship between the number of newly formed neuroblasts and cognitive impairment, with MCI patients exhibiting fewer DCX^+^/PCNA^+^ cells than cognitive normal subjects (Tobin et al., 2019). Another study correlated adult hippocampal neurogenesis with AD and major depressive disorder, which are known to interact reciprocally elevating the risk for one another (Berger et al., 2020). Although several previous researches unsuccessfully attempted to identify adult hippocampal neurogenesis in humans, and then questioned the existence of this process in the adult brain (Paredes et al., 2018; Sorrells et al., 2018), more recent studies demonstrated the existence of this process owing to the application of new methods of tissue sample preservation from postmortem brain, thus allowing a more precise recognition of NSCs, and additionally, the application of this approach to AD patient brains.

## Conclusions

Collectively, literature discussed in this review adds new layers of knowledge on the link between impairment of adult hippocampal neurogenesis and cognitive dysfunction in AD. Specifically, it is reasonable to hypothesize that altered adult hippocampal neurogenesis, due to intracellular accumulation of Aβ and pTau, may have a significant impact on the hippocampal circuitry underlying memory formation, which actively contributes to disease progression. Indeed, data obtained from murine models reminiscent of both FAD and sporadic AD clearly indicate that alterations of neurogenesis (in terms of reduced NSC proliferation, survival of neuroblasts, and functional integration of newly formed neurons) occur before the appearance of memory impairment and that stimuli, increasing hippocampal neurogenesis, ameliorate cognitive functions of AD mice. To date, a correlation between altered hippocampal neurogenesis and AD has been suggested in humans, although the cause–effect relationship between these two processes has not been ascertained yet. Therefore, strategies aimed at restoring and/or boosting adult hippocampal neurogenesis in both normal elderly people and subjects at high risk of AD (e.g., individuals with MCI) could emerge as effective strategies to prevent the onset and/or counteracting the progression of the disease. In this perspective, it is worth mentioning that in mouse models exposed to extremely low-frequency electromagnetic fields a significantly enhanced adult neurogenesis at both hippocampal DG and the subventricular zone has been reported along with memory improvement (Cuccurazzu et al., 2010; Leone et al., 2014; Podda et al., 2014; Mastrodonato et al., 2018).

## Author Contributions

Manuscript was written and revised by RP, DDLP, and CG. All authors approved the final version of the manuscript.

## Conflict of Interest

The authors declare that the research was conducted in the absence of any commercial or financial relationships that could be construed as a potential conflict of interest.
